# Value of Peptide Receptor Radionuclide Therapy as Neoadjuvant Treatment in the Management of Primary Inoperable Neuroendocrine Tumors

**DOI:** 10.3389/fonc.2021.687925

**Published:** 2021-11-12

**Authors:** Marta Opalińska, Anna Sowa-Staszczak, Anna Grochowska, Helena Olearska, Alicja Hubalewska-Dydejczyk

**Affiliations:** ^1^ Nuclear Medicine Unit, Department of Endocrinology, Oncological Endocrinology and Nuclear Medicine, University Hospital, Kraków, Poland; ^2^ Chair and Department of Endocrinology, Jagiellonian University Medical College, Kraków, Poland; ^3^ Department of Radiology, University Hospital, Kraków, Poland

**Keywords:** inoperable neuroendocrine tumors, PRRT, neoadjuvant therapy, NEN, NET (neuroendocrine tumors)

## Abstract

**Introduction:**

Neuroendocrine neoplasms including neuroendocrine tumors (NETs) are often diagnosed as primary disseminated or inoperable. In those cases, systemic extensive therapy is necessary, but radical treatment is unlikely. As described in the literature, in some selected cases, peptide receptor radionuclide therapy (PRRT) may be used as a first-line/neoadjuvant therapy that allows further successful surgery. Such treatment may enable a reduction of total tumor burden or allow a radical treatment which improves the final outcomes.

**Aim:**

This study aims to assess whether neoadjuvant PRRT could be a treatment option for patients with initially unresectable NETs.

**Methods:**

Among the group of 114 patients treated with PRRT between the years 2005 and 2020, in 32 cases, it was the first-line therapy, mainly due to massive disease burden at the time of diagnosis. Among them, nine patients received PRRT as the first-line treatment due to the primary inoperable tumors with the intention of preoperative reduction of the tumor size in order to allow for a surgical treatment.

**Results:**

Neoadjuvant PRRT enabled surgery in four out of nine (45%) patients. Finally, in two out of four cases, the goal (radical surgery) has been achieved.

**Conclusion:**

PRRT may be considered not only as a palliative but also as a neoadjuvant therapy in advanced, somatostatin-positive NETs that were initially inoperable.

## Introduction

Well-differentiated neuroendocrine tumors (NET G1, G2, and G3 according to WHO 2019 classification) are a widely heterogeneous group of malignancies regarding their place of origin, clinical presentation, hormone secretion, tumor growth, and metastases spread rate. A common feature of most NETs is overexpression of somatostatin receptors on their surface, which became the molecular basis for a theranostic approach using somatostatin analogs in the diagnosis and therapy of NETs.

However, in the presence of a localized, non-metastatic disease, surgery is the most effective treatment procedure which can enable complete recovery. In the case of advanced NETs not eligible for surgical treatment, several different antitumor therapeutic options may be used, but a chance for radical treatment is very low (1, 2). Among them, long-acting somatostatin analogs are the first-line treatment in the vast majority of NETs. Nevertheless, in some clinical settings, initial therapy with peptide receptor radionuclide therapy (PRRT) may bring benefits before further treatment. Several clinical trials proved PRRT to be one of the most effective therapeutic options in terms of objective responses in disseminated NET treatment. It has been demonstrated to be effective not only in improving progression-free survival (PFS) but also overall survival (OS) in those patients (3–5). Moreover, in selected cases, it may enable the resection of primarily inoperable tumors (6–8). For that reason, the rationale for the use of PRRT as first-line treatment may be especially valuable in case of extensive disease burden at the time of diagnosis, hormonal syndromes resistant to somatostatin analogs, or a chance for subsequent curative surgery. However, the overall outcome in the abovementioned clinical situations remains completely different.

The purpose of this study was to evaluate if PRRT used as neoadjuvant therapy in patients with NETs may enable radical surgery.

## Materials and Methods

Among a group of 114 patients treated in our center with PRRT between the years 2005 and 2020, 32 of them received PRRT as first-line therapy. Nine of them were qualified for PRRT with the intention of preoperative reduction of the tumor size, which could lead to potential subsequent radical surgery. The “unresectable primary tumor” was defined as extensive large vessel infiltration by neoplastic tissue or tumor invasion to adjacent organs, visualized on preoperative CT scans. All patients referred for preoperative PRRT were consulted by a multidisciplinary team including an oncological surgeon and a radiologist.

In this group, all patients had a histopathological diagnosis of well-differentiated NET according to the European Neuroendocrine Tumor Society–World Health Organization 2010 and 2017 grading system before PRRT, depending on the time of diagnosis. In eight patients, foregut tumors were present [in two in the lungs and in six in the pancreas (pNET)], and one patient was diagnosed with a midgut tumor (small intestine). In two patients, lesions were hormonally active (one insulinoma, one glucagonoma), and in another two, there was a suspicion of single liver metastasis detected in somatostatin receptor imaging (SRI) or computed tomography (CT) scans. Before and after PRRT, all of them were in generally good condition (Karnofsky index over 70%).

All patients qualified for PRRT had a positive result (Krenning scores 3 and 4) of SRI [(99mTc)Tc-octreotide SPECT/CT or (68Ga)Ga-DOTA-TATE PET/CT]. Cytoreductive chemotherapy or long-acting somatostatin analog was not used before PRRT in seven cases. Two patients received chemotherapy prior to PRRT with no response.

In all patients, 3 to 5 cycles of PRRT were applied. [90Y]Y-DOTA-TATE [mean cumulative dose 13.4 GBq ( ± 1.44)] and [90Y]Y/[177Lu]Lu-DOTA-TATE (cumulative dose 14.8 GBq) were applied in eight patients and one patient, respectively. To reduce the radiation dose to the kidneys, as recommended, an infusion of amino acids (arginine and 2.5% lysine) was administered.

The type of radiopharmaceutical used for PRRT depended on PRRT type availability in consecutive years. Routine blood count, liver function, and kidney function were assessed before each therapy cycle and at follow-up visits.

CT was performed 1–3 months prior to PRRT and 4–6 months after PRRT. Multidetector row spiral CT of 2 mm slice thickness and reconstruction increment were used after the administration of non-ionic contrast media. Further follow-up examinations were performed according to the applicable guidelines and the individual clinical course of the disease.

Diameter, volume, and the mean attenuation reduction of each lesion were calculated by CT image processing software.

Tumor response was assessed according to the Response Evaluation Criteria in Solid Tumors (RECIST) 1.1, where the partial response to the therapy is described as ≥30% decrease of the sum of the longest diameters of target lesions, whereas progression is a ≥20% increase of it (**Table 1**). The Choi criteria define objective response as a ≥10% decrease in the sum of tumor diameters or ≥15% decrease in the tumor density on contrast-enhanced CT scan (**Table 1**).

**Table 1:** Definition of radiological responses to therapy according to RECIST 1.1 and Choi criteria.

	RECIST 1.0/1.1	Choi
**Measurement**	**Largest diameter**	**Largest diameter + attenuation**
**Complete response (CR)**	Disappearance of all target lesions	Disappearance of all target lesions
**Partial response (PR)**	At least a 30% decrease in the sum of the greatest unidimensional diameters of target lesions	Decrease in tumor size ≥10% or decrease in tumor density ≥15% on CT
**Disease progression (PD)**	An increase of at least 20% in the sum of the diameters of target lesions	Increase in tumor size ≥10% and does not meet PR criteria by tumor density
**Disease stabilization (SD)**	Does not meet the criteria for CR, PR, or PD	

Response categories were assessed on a subsequent CT scan until the disease progression.

### Statistics

Percentage changes in tumor diameter, volume, and density of tumor mass 1–3 months before and 4–6 months after PRRT were counted as well as the response to PRRT in RECIST 1.1 scale and Choi criteria. Additionally, the percentage of patients who underwent surgery (including complete surgical excision of the tumor) was assessed.

Finally, PFS and OS were calculated. PFS was defined as the time from the first PRRT to radiological or clinical disease progression or death from any cause.

## Results

The group of nine patients (six males and three females) were eligible to the analysis. The mean age of the patients equaled 53.78 years ( ± 14.86, range: 28–78 years).

After the PRRT, the median tumor diameter changed by −1.6 cm (range from −3.7 to 0.3 cm). The median tumor volume decreased by 105.0 cm^3^ (range from −186.2 to 34.7 cm^3^), whereas attenuation decreased by 9.1 HU (range from −17.6 to 17.9 HU). There was no significant difference in the reduction of the tumor diameter, volume, and attenuation between pNET and other (not pNET) lesions (**Table 2**).

**Table 2:** Changes in median (range) of diameter, volume, and attenuation of tumor before and after PRRT in pNET and not pNET patients.

	Median difference of tumor diameter before and after PRRT, cm (range)	Median difference of tumor volume before and after PRRT, cm^3^ (range)	Median difference of tumor attenuation before and after PRRT, HU (range)	Statistical significance
pNET	−0.4 (−3.70 to 0.30)	−7.8 (−186.20 to 34.72)	1.0 (−17.60 to 17.90)	NS
Not pNET	0.0 (−1.47 to 0.00)	−0.1 (−125.94 to −0.1)	−4.2 (−4.40 to 6.10)	NS

pNET, pancreatic NET; not pNET, not pancreatic NET; NS, not significant.

According to RECIST 1.1 criteria, stabilization of the disease (SD) and partial response (PR) were observed in six and one patient, respectively, and progressive disease (PD) was seen in two patients. In two patients, liver metastases described in the initial SRI were not found after PRRT on the follow-up SRI scans, but in one of them, a new SRI negative lesion was detected on CT examination.

According to the Choi criteria counted in eight patients, SD was observed in three, PR in three, and PD in two cases. The correspondence of those two scales was low and the evaluation of the PRRT results was comparable only in 50% of the cases (four patients) (**Table 3**).

**Table 3:** Presentation and radiological and clinical outcomes of the patients.

No.	Gender	Place of primary tumor	Metastases to the liver	Type of PRRT	Change of tumor diameter after PRRT	% change of tumor volume after PRRT	Response to PRRT in RECIST criteria	% change of tumor attenuation after PRRT	Response to PRRT in CHOI criteria	Surgery	R 0	Time to progression after PRRT	Follow-up (months)	Status at last follow-up
1	F	Lung	No	90Y	0%	−0.6%	SD	10.7%	SD	N	N	62.5	62.5	Dead
2	M	Pancreas	No	90Y	−48%	−80.7%	PD (new liver lesion)	3.5%	PD (new liver lesion)	Y	N	3.1	51.8	Dead
3	F	Pancreas	No	90Y	−1%	12%	SD	−25%	PR	N	N	59.5	117.8	Dead
4	M	Pancreas	No	90Y	5%	5%	SD	30%	PD	N	N	0.7	93.4	Dead
5	M	Small intestine	No	90Y	−21%	−60%	SD	−10%	PR	Y	N	65.0	105.6	Dead
6	M	Lung	No	90Y	0%	−3%	SD	−9.0%	SD	N	N	2.3	7.6	Dead
7	M	Pancreas	No	90Y	−30%	−83%	PR	19%	PD	N	N	16.3	48.1	Dead
8	F	Pancreas	Yes	90Y	n/a	n/a	PD	n/a	n/a	Y (hemi-hepatectomy)	Y	3.8	116.2	Alive
9	M	Pancreas	Yes	177Lu/90Y	−8%	−18%	SD	−11%	PR	Y	Y	26.4	26.4	Alive

PD, disease progression; SD, disease stabilization; PR, partial response; R0, surgical resection assessed as radical in histopathology report; Y, yes; N, no; n/a, not available.

The median time of follow-up was 56.9 months (range from 7.8 to 117.7 months). PRRT did not cause clinically important myelotoxicity or nephrotoxicity (CTCAE version 5.0 grades 3 and 4).

Among the whole group of patients, surgery was performed in four cases (45%), but a radical procedure was possible only in two of them (22%). The main cause of renouncement or ineffectiveness of surgery was an infiltration of the large vessels by neoplastic tissue, visualized on CT scans or found during the operation. No surgical complications which could be related to PRRT administration were observed. There was no perioperative mortality.

Two patients who underwent radical surgery are free from disease as of now, one of them for 27.13 months and another for 117.43 months. Both remain in the follow-up group. The assessment of radiological response to PRRT in patient no. 9 (treated radically) differed on the RECIST 1.1 and Choi scales, being SD and PR, respectively (**Table 3**).

In patient no. 8, based on medical documentation, the tumor mass significantly decreased after PRRT, which then enabled surgical intervention. Unfortunately, the CT scan done after PRRT completion was not available.

Among other two patients who underwent incomplete surgery, PFS equaled 8.2 and 72.9 months.

In the group of patients who did not qualify for surgery, the median PFS was 21.5 months (range from 5.6 to 70.1). The median OS for the whole group was 56.9 months (range from 7.6 to 117.7) (**Table 4**). No significant difference in survival time was observed in patients stratified according to primary localization of NET (pNET *vs.* non-pNET).

**Table 4:** Long-term outcome of the patients who underwent PRRT as neoadjuvant therapy.

Features	All patients (*n* = 9)
**Disease progression up to 6 months after PRRT**	2
**Time to progression, months; median (range)**	21.5 (5.8–64.7)
**Overall survival, months; median (range)**	56.9 (7.6–116.7)
**Surgeries after PRRT, *n***	4
**Radical surgeries after PRRT, *n***	2

## Discussion

Neoadjuvant therapy is an initial therapy which may be given to shrink the neoplastic tumor and enable further surgical intervention. It is widely used in different types of cancers including breast, pancreatic, and others, but not common in NETs due to usually large tumor burden at diagnosis.

According to current ENETS guidelines, various systemic therapies are available for locally advanced, metastatic, and progressive gastroenteropancreatic neuroendocrine tumors (GEP-NETs) (1). Long-acting somatostatin analog therapy is applied as a first-line treatment in the presence of somatostatin receptor (SSTR) expression at molecular imaging. The second- or third-line therapy regimens include chemotherapy with capecitabine and temozolomide (CAPTEM), PRRT, protein kinase inhibitors, streptozocin-based chemotherapy, or locoregional therapies, usually liver-directed (2, 3). PRRT is effective independently of the type of beta minus emitter (Y-90/Lu-177) or somatostatin analog (TATE/TOC) being used (9, 10). Moreover, PRRT efficacy is high although the schemes of therapy and the use of specific radionuclide differ between centers. The direct effectiveness of PRRT in comparison with other types of therapy regimens is planned to be evaluated on the basis of ongoing or future clinical trials including comparison of PRRT to everolimus in progressing G1 and G2 GEP-NETs (COMPETE, ClinicalTrials.gov Identifier: NCT03049189) or to everolimus, FOLFOX, and CAPTEM in aggressive G2 and G3 GEP-NETs (COMPOSE, ClinicalTrials.gov Identifier: NCT04919226).

Among other treatment possibilities, temozolomide has shown antitumor activity in pNETs either as monotherapy or in combination with capecitabine (CAPTEM) or bevacizumab. The objective response rates ranged from 33% (11) to 70% (12), with the highest response rates in studies using CAPTEM. However, the use of CAPTEM regimen in patients with localized pNEN stratified by grade and neoadjuvant or adjuvant therapy in comparison to somatostatin analog was associated with poorer OS (13), which raises doubts about the potential use of CAPTEM as first-line therapy even with the intention of using it as neoadjuvant therapy. In one of the studies, neoadjuvant CAPTEM regimen with or without radiation has been successfully applied in six pNETs with borderline resectable disease. All patients had radiological evidence of tumor regression after neoadjuvant treatment (two PR and four SD stabilization), and all of them could undergo successful resection of the primary tumor with negative margins in four out of six patients (14).

Throughout the 15 years of PRRT treatment in our center, we used both Y-90 and Lu-177 separately or as a tandem therapy combining Y-90 and Lu-177 with an activity ratio of 1:1. In all types of PRRT schemes, positive results were observed after the use of PRRT as first- or second-line therapy. In very few cases, PRRT was administered in an attempt to reduce the baseline tumor size with an intention of further radical surgical treatment. This approach offers hope for complete recovery which is not likely achievable with other forms of systemic treatment. Until now, there are only a few publications summarizing the use of PRRT as neoadjuvant therapy in NET patients, and a significant number of them relate to small groups of patients and case reports. The publication describing the largest group of patients who underwent neoadjuvant PRRT shows an encouraging rate of successful surgeries even in 31% of patients (9 out of 29 cases) (15). In our material, the rate of successful surgeries after PRRT was slightly lower (22%) in comparison with the abovementioned publication, but the rate of complete recoveries still appears inspiring enough to consider such treatment in selected cases. It is worth emphasizing that among our patients, there was one case who presented single liver metastasis on CT/SRI scans prior to PRRT with no evidence of hepatic lesions on both CT and SRI scans after PRRT treatment. Similar results, including the cure of liver metastases by PRRT, were described in the past by a few authors (16–19). Those observations also encourage considering PRRT as neoadjuvant therapy even in the presence of a single liver metastases, especially if they are poorly available for locoregional treatment.

The second potential advantage of the use of PRRT at the beginning of treatment is a significant decrease of total tumor burden. This fact was clearly demonstrated mainly for 177Lu-DOTA-TATE therapy in one randomized trial (NETTER-1 trial) (3) and several non-randomized trials (20). According to a meta-analysis done on patients with disseminated pancreatic NETs, the pooled median PFS after PRRT was 25.7 months (95% CI: 18.9–32.4 months) and was better than in patients treated with everolimus [PFS 14.7 months (95% CI: 11.2–18.1 months)] (21), which is recommended as second-line treatment in disseminated pNETs. In our group, median PFS (in a corresponding group of patients who did not undergo surgery) was 21.5 months (range 5.6–70.1). The results obtained in our group are significantly better, which probably results from the selection of patients with a chance of radical surgery, i.e., with a relatively small disease burden, without multiple metastases. The PFS increase additionally encourages the use of PRRT at the beginning of treatment, especially if there is initially high tumor burden and when prolongation of PFS (less probable to achieve with the use of other systemic therapy) may be considered as an added benefit.

**Figure 1:**
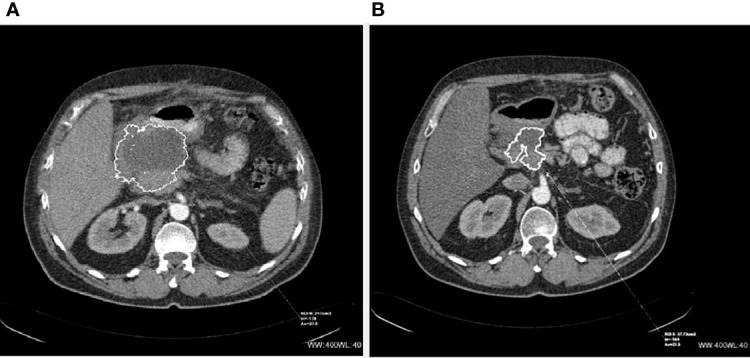
CT scans of inoperable (before and after PRRT) NET of the pancreas: **(A)** before PRRT and **(B)** 4 months after 4 cycles of 90Y-DOTA-TATE.

**Figure 2:**
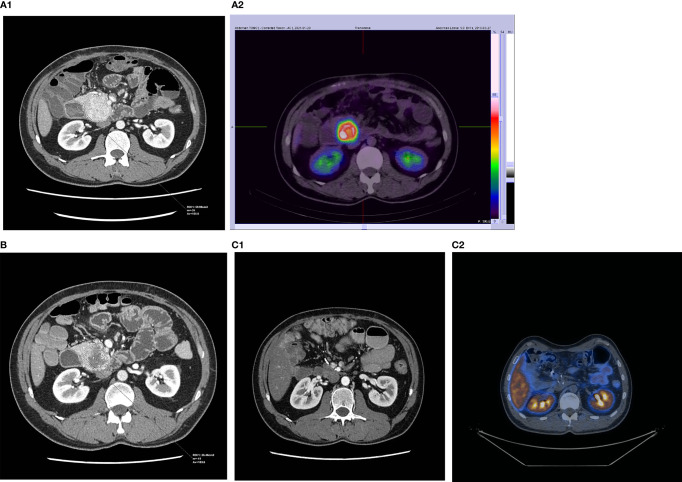
CT and SRI scans of a patient with successfully operated NET of the pancreas: **(A1, 2)** before PRRT, **(B)** 3 months after 4 cycles of 177Lu/90Y-DOTA-TATE (only CT), and **(C1, 2)** after complete tumor removal.

The results of PRRT assessed as disease regression, stabilization, or progression depend on the radiological method used for the evaluation of response to therapy. The most common methods used for that purpose are RECIST, Southwest Oncology Group (SWOG), or Choi criteria. RECIST scale (1.0 and more common nowadays 1.1) is already a radiological gold standard for the assessment of tumor response to cytoreductive treatment for different malignancies. However, this scale is hardly efficient in the validation of neoplasms with relatively slow growth (22). The main weakness of this scale is that it measures only the longest diameters of all selected target lesions, while the linear diameter does not vary sufficiently to correct estimation of total lesion volume. For this reason, scales such as the Choi criteria, which try to also take into account changes in the radiological density of lesions (reduction of attenuation, weaker contrast enhancement as the effect of neoplastic tissue necrosis), were created. Those scales are considered to be more useful in tumors with relatively slower growth. In our material, the response of seven out of nine patients (assessed as a PR or SD) to the treatment fits into one of those scales: seven out of nine in the RECIST scale and five out of eight into the Choi criteria. However, the same type of response in both scales was seen in four cases showing relatively poor compatibility (50%) of both of those rating systems. Another side of the imperfection of those scales is seen in the example of a patient with a pancreatic NET producing insulin. After [90Y]Y/[177Lu]Lu-DOTA-TATE treatment, the tumor diameter decreased by 8%, its volume by 18% and attenuation decreased by 11%. It allowed us to assess the response to therapy as SD and PR according to RECIST and CHOI criteria respectively (**Figures 1**, **2**). However, the reduction of tumor size and the decrease of tumor vascular involvement enabled curative surgery, confirming that neither of those radiological tools is highly effective in the preliminary assessment of PRRT efficacy nor does it predict a clinical outcome (patient was radically operated) (**Figures 1**, **2**). It implies that it is very difficult to indicate, before qualification for PRRT, whether or not the patient will respond to the therapy and what the maximal tumor size change will be and whether PRRT may be considered as a neoadjuvant therapy. Moreover, in our work, the reduction of tumor volume was significant in many cases, but in two cases, we observed disease progression which means that PRRT did not always bring about the expected outcome. Finally, we also counted the percentage of tumor size shrinkage after PPRT, and we found that the response to therapy in both groups (pNETs *vs.* non-pNETs) was similar. Unfortunately, the cardinality of the group studied in our work was too small to draw unequivocal conclusions as to whether the use of different PRRT types and schemes brings about the same results. Among many radiological and clinical features, only negative results of [18F]FDG PET/CT examination, histopathological grading (23), and to some extent the good SSTR expression on SRI (24) are widely known indicators of prognosis in NET patients. Nevertheless, none of those parameters are confirmed as factors influencing PRRT to be a neoadjuvant therapy.

It should also be considered that the use of different radionuclides (Y-90 and Lu-177 or mixed Y-90/Lu-177 having different radiation lengths and energies) may have an impact on the final outcome of the treatment. However, there are currently no studies comparing the different types of radionuclides used for PRRT.

Although we have not found an association between clinical and radiological features which could be helpful in proper patient selection for neoadjuvant PRRT, it is worth noting the possibility of the multigenomic blood mRNA biomarker (NETest) and PRRT predictive quotient (PPQ) use. PPQ had been evaluated as a predictor of PRRT response in 97%. NETest accurately monitors PRRT response and is an effective surrogate marker of PRRT radiological response (25). Perhaps, it will be possible to use those parameters, facilitating the selection of patients who have a greater chance for radical surgery after neoadjuvant therapy.

Despite the lack of serious adverse events in our cohort, PRRT may be associated with the risk of short- and long-term side effects. Most side effects are connected directly with myelosuppression reversible and rather dose-limiting, but the problem of long-term complications remains crucial due to the expected long-time survival in radically treated patients. The most important long-term complications include myelodysplastic syndrome, acute myeloid leukemia, or bone marrow aplasia with the median latency period at diagnosis about 41 months (26). The prevalence of those severe, delayed adverse hematological events is estimated at 1.4%–4% (27, 28). In case of PRRT radiopharmaceuticals labeled with 90Y, kidney-related toxicity should also be considered (29). Some of those toxicities may be limited by proper dosimetry.

## Conclusions

- In some cases of SSTR-positive NETs, PRRT used as a first-line treatment may cause significant tumor size reduction, which enables radical surgical intervention. In other cases (in majority of the patients), the benefits include reduction of total tumor burden and long-term stabilization of the disease according to RECIST criteria.
- To date, there are no clinical or radiological features (except high tumor burden) that give a fully unambiguous answer to the question of whether PRRT may allow for radical surgical treatment.
- All PRRT regimens can be considered as a useful therapy for somatostatin receptor-positive NETs, including the application of PRRT as a neoadjuvant therapy in primary inoperable tumors. Currently, there are no data indicating which PRRT regimen (177Lu, 90Y/177Lu, 90Y; TATE/TOC) and schemes could be most effective.
- PRRT was clinically well tolerated and did not interfere with the subsequent surgical or oncological treatment.

## Data Availability Statement

The original contributions presented in the study are included in the article/supplementary material. Further inquiries can be directed to the corresponding author.

## Ethics Statement

The study protocol was approved by the Local Ethics Committee of the Jagiellonian University in Krakow (reference number: 1072.6120.32.2021). The patients/participants provided their written informed consent to participate in this study.

## Author Contributions

MO: data collection, imaging review, data analysis, manuscript drafting, manuscript editing and approval, and study coordination. AS-S: data collection, imaging review, data analysis, manuscript drafting, and manuscript editing and approval. AG: data collection, imaging review, manuscript drafting, and manuscript editing and approval. HO: data collection and manuscript editing and approval. AH-D: data collection, imaging review, data analysis, manuscript drafting, and manuscript editing and approval. All authors contributed to the article and approved the submitted version.

## Conflict of Interest

The authors declare that the research was conducted in the absence of any commercial or financial relationships that could be construed as a potential conflict of interest.

## Publisher’s Note

All claims expressed in this article are solely those of the authors and do not necessarily represent those of their affiliated organizations, or those of the publisher, the editors and the reviewers. Any product that may be evaluated in this article, or claim that may be made by its manufacturer, is not guaranteed or endorsed by the publisher.
